# Sequelae of premature loss of lower permanent molars on developing occlusion during the mixed dentition period—A radiographic evaluation

**DOI:** 10.1002/ccr3.2866

**Published:** 2020-04-22

**Authors:** Nandini Kumari Katta, Sudheesh Kakkunath Mani

**Affiliations:** ^1^ Department of Paediatric Dentistry AIMST University Bedong Malaysia; ^2^ Department of Oral and Maxillofacial Surgery AIMST University Bedong Malaysia

**Keywords:** extraction, malocclusion, molar, permanent, premature

## Abstract

When planning extraction of teeth with poor prognosis especially lower first permanent molars, it is important to consider the timing of tooth removal and its effect on future occlusion to avoid interventions.

## Graphical abstract

Timing of mandibular first molar extraction if not planned well can lead to complications like space loss, tilting, and lingual rolling of mandibular second molar and supra‐eruption of maxillary first molar. Hence, the timing of mandibular first molar extraction in mixed dentition must be planned according to the status of the existing dentition to avoid any complications.

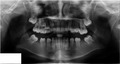

## 1 CASE PRESENTATION

**FIGURE 1:**
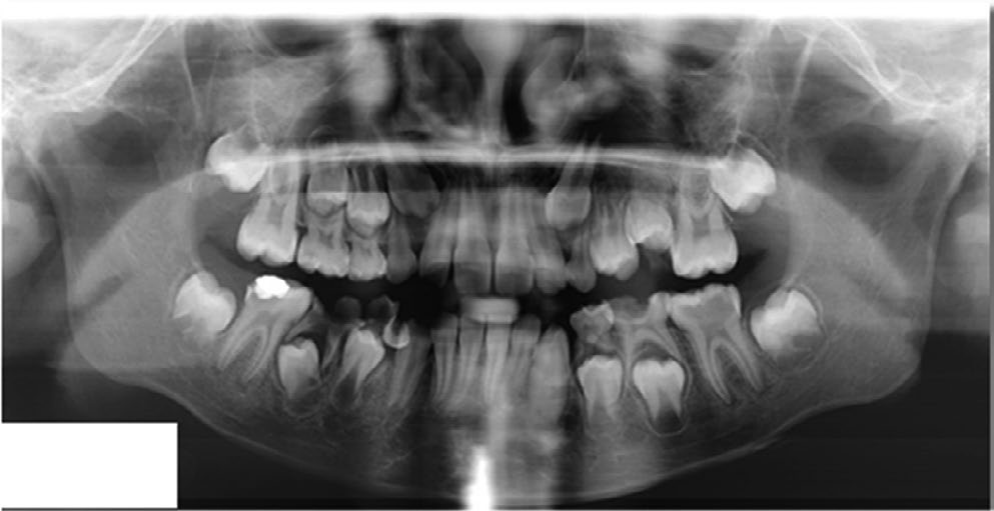
Orthopantomogram of a 9‐y‐old female patient before extraction of the teeth 36, 46

**FIGURE 2:**
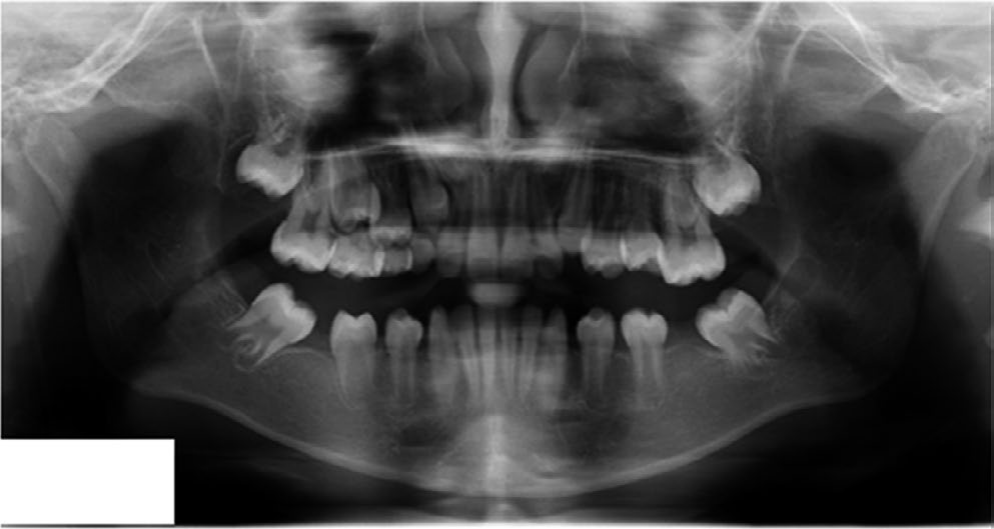
Orthopantomogram of the same patient taken a year after the first visit shows the Sequelae of premature extraction of the teeth 36, 46

**FIGURE 3:**
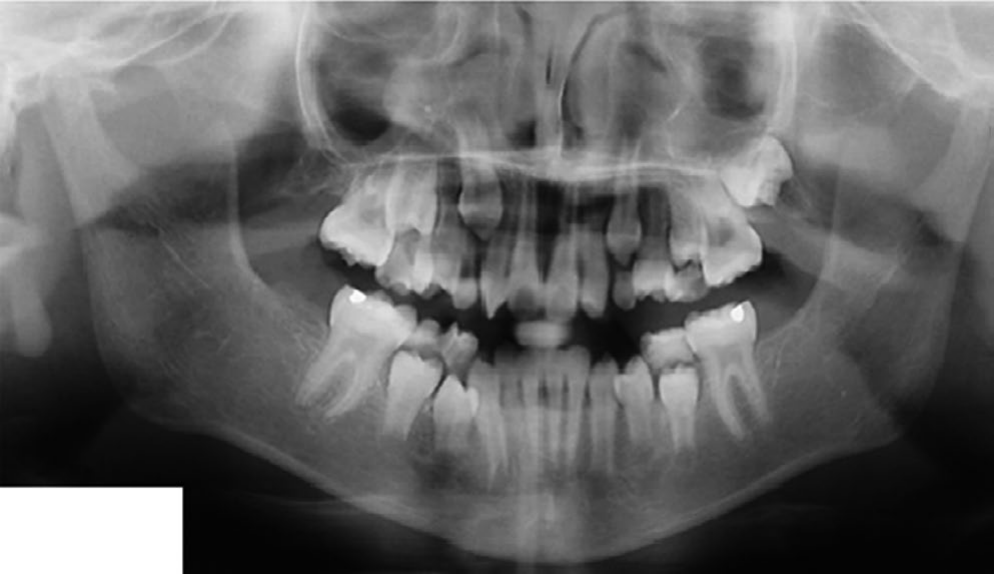
Orthopantomogram of a 10‐y‐old female patient showing congenitally missing teeth 17, 37, 47 and absent 3rd molar tooth buds

Orthopantomogram (OPG) of a 9‐year‐old female patient (Figure 1) before extraction of the teeth 36, 46 shows multiple decayed deciduous teeth, deep caries with tooth 36, restoration with underlying secondary caries in tooth 46, and periapical radiolucency with 36 and 46. Figure 2 OPG of the same patient taken a year after the first visit shows the sequelae of premature extraction of the teeth 36, 46 as mesial drifting of teeth 37, 47, loss of space, supraeruption of teeth 16, 26, generalized spacing in the lower arch and disturbed occlusion. Referral was done for further orthodontic opinion. Figure 3 OPG of a 10‐year‐old female patient reported for a routine dental checkup shows class I molar relationship with a cantered dental midline, peg laterals, primary teeth with preshedding mobility, mesially rotated lower right first premolar, amalgam restoration with teeth 36, 46, congenitally missing teeth 17, 37, 47, and absent 3rd molar tooth buds. The occlusion was undisturbed with no masticatory issues. This case in contrast to the case 1 had congenitally missing second permanent molars with undisturbed occlusion emphasizing on the importance of first permanent molars in the developing occlusion.^1^, ^2^

When planning extraction of teeth with poor prognosis especially lower first permanent molars, it is important to consider the timing of tooth removal and its effect on future occlusion to avoid unnecessary interventions.

## CONFLICT OF INTEREST

There are no conflicts of interest.

## AUTHOR CONTRIBUTIONS

NKK: wrote the manuscript. SKM: involved in proof reading.
